# Microbiome alterations in women with pelvic organ prolapse and after anatomical restorative interventions

**DOI:** 10.1038/s41598-023-44988-6

**Published:** 2023-10-16

**Authors:** Myungshin Kim, Seungok Lee, Hoon Seok Kim, Mi Yeon Kwon, Jaeeun Yoo, Min Jeong Kim

**Affiliations:** 1 Departments of Laboratory Medicine, Seoul St. Mary’s Hospital, College of Medicine, The Catholic University of Korea, Seoul, Republic of Korea; 2 Departments of Laboratory Medicine, Incheon St. Mary’s Hospital, College of Medicine, The Catholic University of Korea, Seoul, Republic of Korea; 3 Departments of Clinical Medicine Research, Bucheon St. Mary’s Hospital, The Catholic University of Korea, Seoul, Republic of Korea; 4 Departments of Obstetrics and Gynecology, Bucheon St. Mary’s Hospital, College of Medicine, The Catholic University of Korea, Seoul, 14647 Republic of Korea

**Keywords:** Clinical microbiology, Microbiology, Diseases, Health care, Molecular medicine, Urology

## Abstract

Pelvic organ prolapse (POP) is a benign gynecological disease in which the pelvic organ descends into the vagina and causes voiding, and defecatory dysfunction, mainly occurs in older women. This study aimed to investigate the vaginal microbiome of POP and associated changes after anatomical restorative pessary or reconstructive pelvic operation. We analyzed the vaginal microbiome using 16S ribosomal RNA gene sequencing and compared the results among patient groups with POP, pessary, and postoperation. We also measured 10 inflammation-related cytokines in vaginal swab samples using multiplex immunoassay. In pelvic organ prolapse, vaginal community status type IV was the most prevalent, which showed a low abundance of *Lactobacillus* with increased diversity and abundance of anaerobic species. The alpha diversity of species richness was highest in the POP group. The beta diversity distance differed significantly between the three groups (*p* = 0.001). While human intestinal taxa-associated bacteria were reduced after pessary or operation, vaginitis-associated bacterial composition was altered but vaginal microbiome homeostasis was not improved. IFN-γ, IL-10, IL-12p70, IL-1β, IL-4 and TNF-α levels increased in the pessary group. Therefore, in addition to anatomical restorative treatment, supplementary treatment focusing on the recovery of the vaginal microbiome may be needed to maintain the health of gynecological organs in old age.

## Introduction

Pelvic organ prolapse (POP) is a benign gynecological condition characterized by pelvic organ descent into the vagina causing vaginal bulge and pressure, as well as voiding, and defecatory dysfunction^1,2^. According to the National Health and Nutrition Examination Survey, approximately 3% of women in the United States report POP symptoms^3^ and peak incidence of POP symptoms is in women aged 70 to 79 years^2^. In Korea^2^, POP surgery peaks in women approximately 70 years old and the use of pessary has increased dramatically in women older than 65 years. POP is not well diagnosed until symptoms appear, the prevalence of POP based on reported symptoms was much lower (3 to 6%) than the prevalence identified by examination (41 to 50%)^4^. As an aging society, diseases such as pelvic organ prolapse, due to life extension, are expected to increase gradually.

Many women experience POP symptoms as they get older, but there is no standardized diagnosis, management, or treatment yet. POP treatment options focus on restoring anatomical structure and includes: an effective reversal non-surgical vaginal pessary and permanent vaginal reconstructive operation^1^. The patient's age, medical condition, and preference for surgery, are the factors considered when deciding on a treatment option. For older patients with an underlying disease, conservative non-surgical treatment is preferred.

Pessary usage or reconstructive operation results in symptom improvement and although pessary success rates range from 77 to 92%^5^, continuous use could cause intravaginal microbiome disruption and mucosal damage, which results in increased discharge and abnormal genital bleeding^6,7^. Little is known about the changes in intravaginal microbiome in pessary users, therefore therapeutic efficacy trials to renovate the vaginal microbiome is rare. Vaginal reconstruction is a surgical procedure that aims to reconstruct the vaginal canal and its surrounding tissues^8^. However, POP operation can result in complications, including rectovaginal or vesicovaginal fistula, ureteral injury, shortened vagina, recurrent pelvic organ prolapse (6–30%)^9^, voiding dysfunction, and pelvic pain^1^. Disturbed vaginal microbiota refers to an imbalance in the normal microbial composition within the vagina. In a healthy state, the vaginal microbiota is dominated lactobacilli, which help maintain a slightly acidic environment and provide protection against potential pathogens. This balanced state is crucial for vaginal health. The protruding pelvic organ could change the vaginal microbiome by friction and external exposure and pessaries more easily caused intravaginal mucosal damage and also potentially disturbed the microbiome more easily^6^. Research on this topic is limited and the exact changes that occur are still not well understood in pelvic organ prolapse.

In this study, we aimed to evaluate and compare vaginal microbiome of patients with POP, pessary treatment, and pelvic reconstruction operation using 16S rRNA sequencing, and to assess the relationship of inflammation-related cytokines in the vaginal swab samples in the prolapse, pessary, and postoperative groups using multiplex immunoassay.

## Results

### Patient demographics

Seventy Korean women were enrolled in this study with a mean age of 71.1 ± 9.9 years and a body mass index (BMI) of 25.2 ± 3.1 kg/m^2^. The clinical characteristics of patients with POP are summarized and displayed using descriptive statistics in Table 1. The average age of the prolapse, pessary and postoperative groups were 68.0 ± 9.7, 79.7 ± 5.9 and 69.6 ± 8.7 years, respectively. Based on the POP-Q system, majority of the participants met the criteria for stage II and III POP (n = 48, 81.4%), and urological symptoms (frequency, urgency) were noted in 28 participants (47.5%). Underlying medical diseases (diabetes mellitus, hypertension, cerebrovascular accident, and thyroid disease) were present in 56 patients (80%). The underlying disease was higher in the pessary group, but there was no statistical difference (94.1%, *p* = 0.13).

**Table 1:** Characteristics of POP patients (Prolapse group (control), Pessary group, and Postoperative group).

Characteristics	Total	Prolapse group	Pessary group	Postoperative group	*P*-value
Number	70	42	17	11	
Mean age (years)	71.1 ± 9.9	68.0 ± 9.7	79.7 ± 5.9	69.6 ± 8.7	0.00008^a^
POP stage (n)					0.90^b^
Stage I	2	2	0	Not checked	
Stage II	21	14	7	Not checked	
Stage III	27	20	7	Not checked	
Stage IV	9	6	3	Not checked	
Not checked	11	0	0	11	
Height (cm)	151.0 ± 6.1	151.9 ± 5.7	149.5 ± 6.7	149.6 ± 6.8	0.29^a^
Weight (kg)	57.5 ± 8.6	58.7 ± 8.1	55.2 ± 9.9	56.1 ± 8.1	0.34^a^
BMI (Kg/m^2^)	25.2 ± 3.1	25.4 ± 3.0	24.6 ± 3.6	25.1 ± 3.2	0.68^a^
Urological symptoms^c^ (frequency, urgency %)	0.26^b^				
Yes	28 (47.5)	22 (52.4)	6 (35.3)	Not checked	
No	31 (52.5)	20 (47.6)	11 (64.7)	Not checked	
Underlying medical diseases (DM, HTN, CVA Thyroid disease)	0.13^b^				
Yes	56 (80.0)	33 (78.6)	16 (94.1)	7 (63.6)	
No	14 (20.0)	9 (21.4)	1 (5.9)	4 (36.4)	

*POP* pelvic organ prolapse; *DM* diabetes mellitus; *HTN* hypertension; *CVA* cerebrovascular accident.

^A^one-way ANOVA.

^b^Fisher’s exact test.

^c^Data for 59 women are shown (results for 11 women in postoperative group).

### Relationship of inflammation-related cytokines and prolapse, pessary and postoperative groups

**Figure 1:**
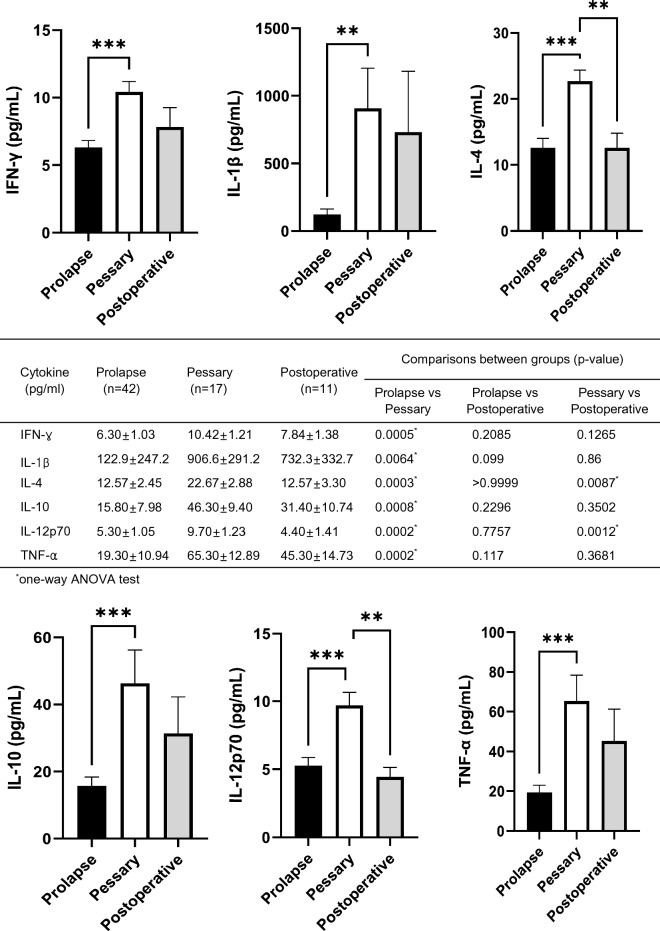
Inflammasome-dependent Pathways. According to the Pearson correlation score, components belonging to the inflammasome complex were significantly (FDR *p* < 0.001) correlated with the POP group (prolapse, pessary, and postoperative). The association was calculated using the observation_metadata_correlation.py script on rarefied otu_table.biom (5000 reads/sample). The levels of IFN-γ, IL-1β, IL-4, IL-10, IL-12p70, and TNF-α varied among groups. Comparisons were performed using one-way analysis of variance. When a significant p value was observed (*p* less than 0.05), a multiple comparison test was used to determine which groups were different. Data are shown as the mean value ± standard error of the mean (SEM). Pearson’s scores for each bacterial species are shown in parentheses. FDR, false discovery rate. **p < 0.05, ***p < 0.001.

IFN-γ (prolapse (6.30 ± 1.03 pg/mL) versus pessary (10.42 ± 1.21 pg/mL), *p* = 0.0005), IL-1β (prolapse (122.9 ± 247.2 pg/mL) versus pessary (906.6 ± 291.2 pg/mL), *p* = 0.0064), IL-4 (prolapse (12.57 ± 2.45 pg/mL, *p* = 0.0003), postoperative (12.57 ± 3.30 pg/mL, *p* = 0.0087) versus pessary (22.67 ± 2.88 pg/mL)), IL-10 (prolapse (15.80 ± 7.98 pg/mL) versus pessary (46.30 ± 9.40 pg/mL), *p* = 0.0008), IL-12p70 (prolapse (5.30 ± 1.05 pg/mL, *p* = 0.0002), postoperative (4.40 ± 1.41 pg/mL, *p* = 0.0012) versus pessary (9.70 ± 1.23 pg/mL)), and TNF-α (prolapse (19.30 ± 10.94 pg/mL) versus pessary (65.30 ± 12.89 pg/mL), *p* = 0.0002) were statistically higher in the pessary group (Fig. 1).

### Overall sequence output

The total valid reads from patient sample (n = 70) 16S rRNA gene sequencing ranged from 22,685 to 143,510 (median = 39,766) determined after quality control (low-quality amplicons, non-target amplicons, and chimeras were removed). The average read length was 418 ± 5 bp per sample. The number of OTUs in the samples ranged from 65 to 468 (median, 152), and the number of species found ranged from 30 to 409 (median, 135 The average percentage of valid reads identified at the species level was 98.4 ± 4.4% (mean ± SD).

### Differences in the vaginal Microbiome Taxonomic Profile (MTP) among prolapse, pessary and postoperative groups

**Figure 2:**
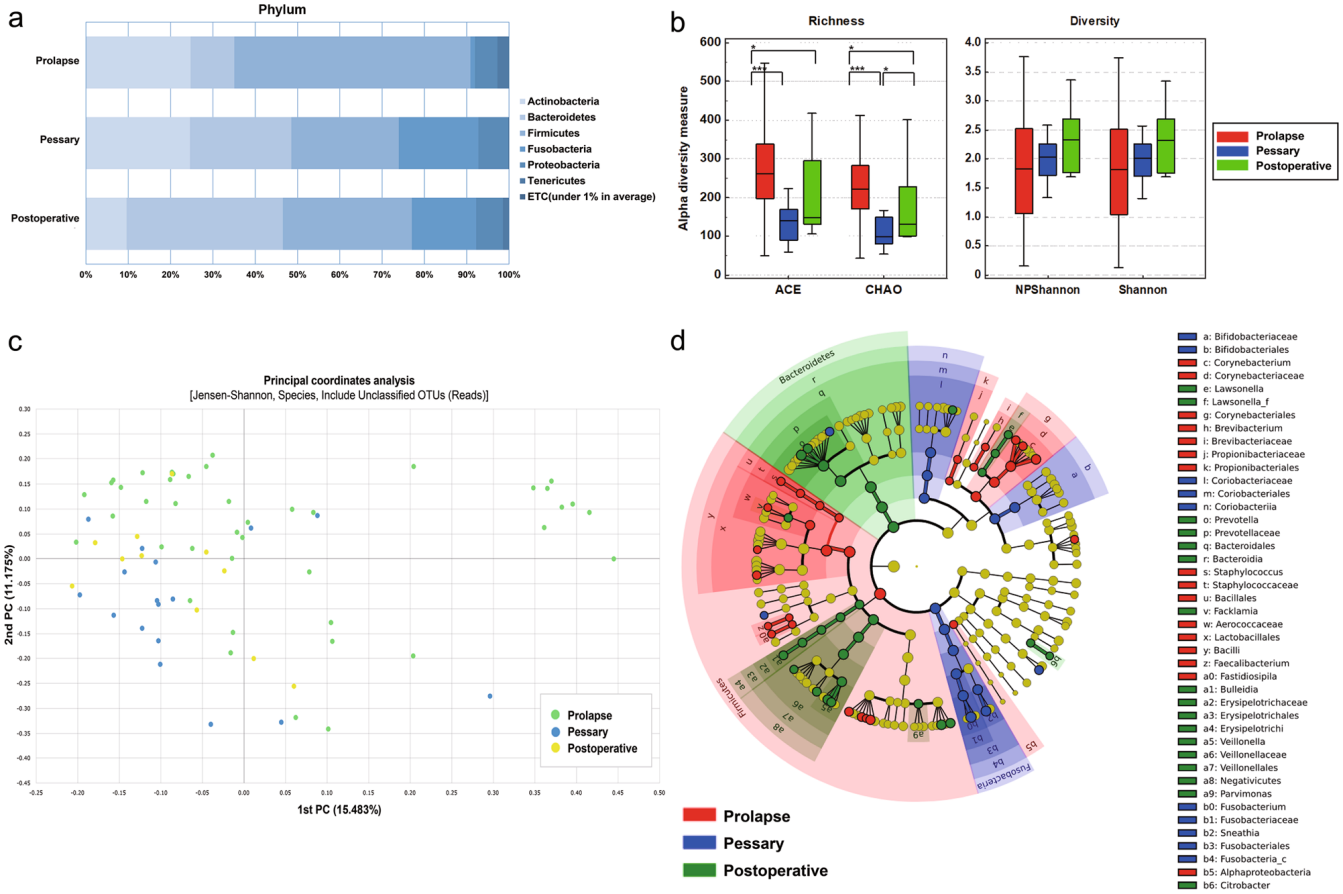
Differences in the vaginal MTP between prolapse, pessary and postoperative groups. (**a**) Relative abundances (%) at the phylum level between the prolapse (n = 42), pessary (n = 17), and postoperative (n = 11) groups. (**b**) Alpha diversity indices showed statistical differences in species richness (ACE and Chao) between the three groups, but there were no statistical differences in diversity (NPShannon and Shannon). Non-parametric test, ^*^p < 0.05, ^**^p < 0.01, and ^***^p < 0.001. (**c**) Principal Coordinate Analysis (PCoA) plots based on Jensen-Shannon beta diversity distances at the species level. *p* = 0.002 for the prolapse and pessary group, *p* = 0.003 for the prolapse and postoperative group, and *p* = 0.548 for the pessary and postoperative group by pair-wise PERMANOVA with 999 permutations. (**d**) Linear Discriminant Analysis Effect Size (LEfSe)-based cladogram showing the differential phylogenic distribution of the bacterial taxa related to the prolapse (red), pessary (blue), and post-operative (green) groups. The taxonomic names of the phyla are indicated; and class, order, family, and genus are abbreviated.

The average taxonomic composition (%) at the phylum level in the three study groups is shown in Fig. 2a. In the prolapse group, Firmicutes (55.8%) was highest, followed by Actinobacteria (24.7%), Bacteroidetes (10.3%), and Fusobacteria (1.0%). In the pessary group, the same phyla were found but in similar proportions: In the pessary group, phyla of Firmicutes (25.4%), Actinobacteria (24.6%), Bacteroidetes (23.9%), and Fusobacteria (18.8%) were found in similar proportions. In the postoperative group, Bacteroidetes (36.9%) was highest, followed by Firmicutes (30.5%), Fusobacteria (15.2%), and Actinobacteria (9.6%). The relative abundance (%) of Firmicutes in the prolapse group was the highest of the three groups (*p* = 0.0024 by Kruskal–Wallis test, with medians of 61.4% for prolapse, 18.6% for pessary, and 24.6% for postoperative, respectively).

Alpha diversity indices showed statistical differences in species richness among the three groups (ACE: median 262, 141, and 147 (*p* < 0.0001) and Chao: median 222, 99 and 132 (*p* < 0.0001), in the prolapse, pessary and postoperative groups, respectively). These results indicated that species richness was the highest in the prolapse group (Fig. 2b).

PCoA plots based on the Jensen-Shannon index, at the species level, is shown in Fig. 2c. The beta diversity distance showed a significant difference among the three groups by PERMANOVA with 999 permutations (*p* = 0.0001). In addition, the significance of beta diversity according to PERMANOVA results was p = 0.002 between the prolapse and pessary groups, *p* = 0.003 between the prolapse and postoperative groups, and *p* = 0.548 between the pessary and postoperative groups. The results revealed that the taxonomic composition of the prolapse group was different from that of the pessary and postoperative groups.

The LEfSe-based Cladogram displayed differential phylogenic distribution of the bacterial taxa associated with each groups (Fig. 2d, Supplmentary Table 1).

Briefely, in the prolapse group, the phylum Firmicutes, and the lower taxa including the genus *Staphylococcus*, *Faecalibacterium* and *Fastidiosipila* were significantly enriched. In the Pessary group, the phylum Fusobacteria, and the lower taxa, including the genus *Fusobacterium* and *Sneathia* were significantly enriched. In the postoperative group, the phylum Bacteroidetes, and the lower taxa including the genus *Prevotella* were significantly enriched.

In the Prolapse group, according to the POP-Q system (level of introitus of uterine descent), there were two patients in stage I, 14 in II, 20 III, and six patients in stage IV. However, Alpha diversity indices showed no statistical differences in species richness (*p* = 0.6921 for ACE and *p* = 0.6272 for Chao by Kruskal–Wallis test) or species diversity (*p* = 0.0867 for NPShannon and *p* = 0.0867 for Shannon by Kruskal–Wallis test) according to the POP-Q system. In addition, there were no statistical differences in beta diversity distance, according to the POP-Q system (*p* = 0.212) in the prolapse group.

### Community state type (CST) groups based on the dominant *Lactobacillus* species

**Figure 3:**
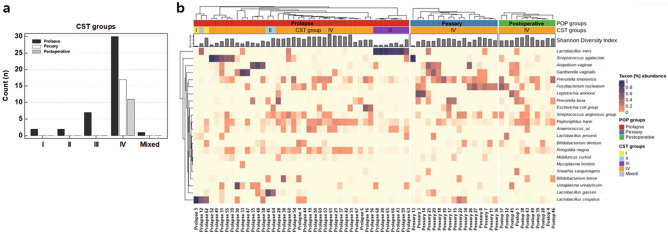
Community state type (CST) groups based on the dominant *Lactobacillus* species. (**a**) Frequency distribution of prolapse (n = 42), pessary (n = 17), and postoperative groups (n = 11). (**b**) Heatmap of the relative abundance of 22 representative microbial taxa in the vaginal microbiota of all 70 cases (color key indicates taxon % abundance). The Shannon index shows the species diversity and evenness for each sample.

The CST group was classified according to the predominant *Lactobacillus* species (Fig. 3a). Overall, CST group IV (low abundance of *Lactobacillus* and increased diversity and abundance of anaerobic species) was most prevalent (82.9%, 58/70). In the prolapse group, the CST IV was 71.4% (30/42), followed by 16.7% (7/42) for *L. iners*-dominant CST group III, 4.8% (2/42) for *L. crispatus*-dominant CST I, 4.8% (2/42) for *L. gasseri*-dominant CST group II, 2.4% (1/42) for mixed CST group with co-dominance of two *Lactobacillus* species. The pessary (17/17) and postoperative groups (11/11) had only CST group IV.

The relative abundance of representative microbial taxa (22) in the vaginal microbiota in all three groups is displayed on a heatmap (Fig. 3b). The Shannon index, which indicates species diversity and evenness for each sample, was relatively high in CST group IV.

In addition, we compared the relationship between CST groups and 10 cytokines using one-way ANNOVA to further investigate the relationship between the microbiota and cytokines. As a result, there were no cytokines that were significantly different from CST group I (n = 2), group II (n = 2), group III (n = 7), group IV (n = 58) and mixed (n = 1) (data not shown).

### Relative abundance of the selected microbial taxa among prolapse, pessary and postoperative groups

**Figure 4:**
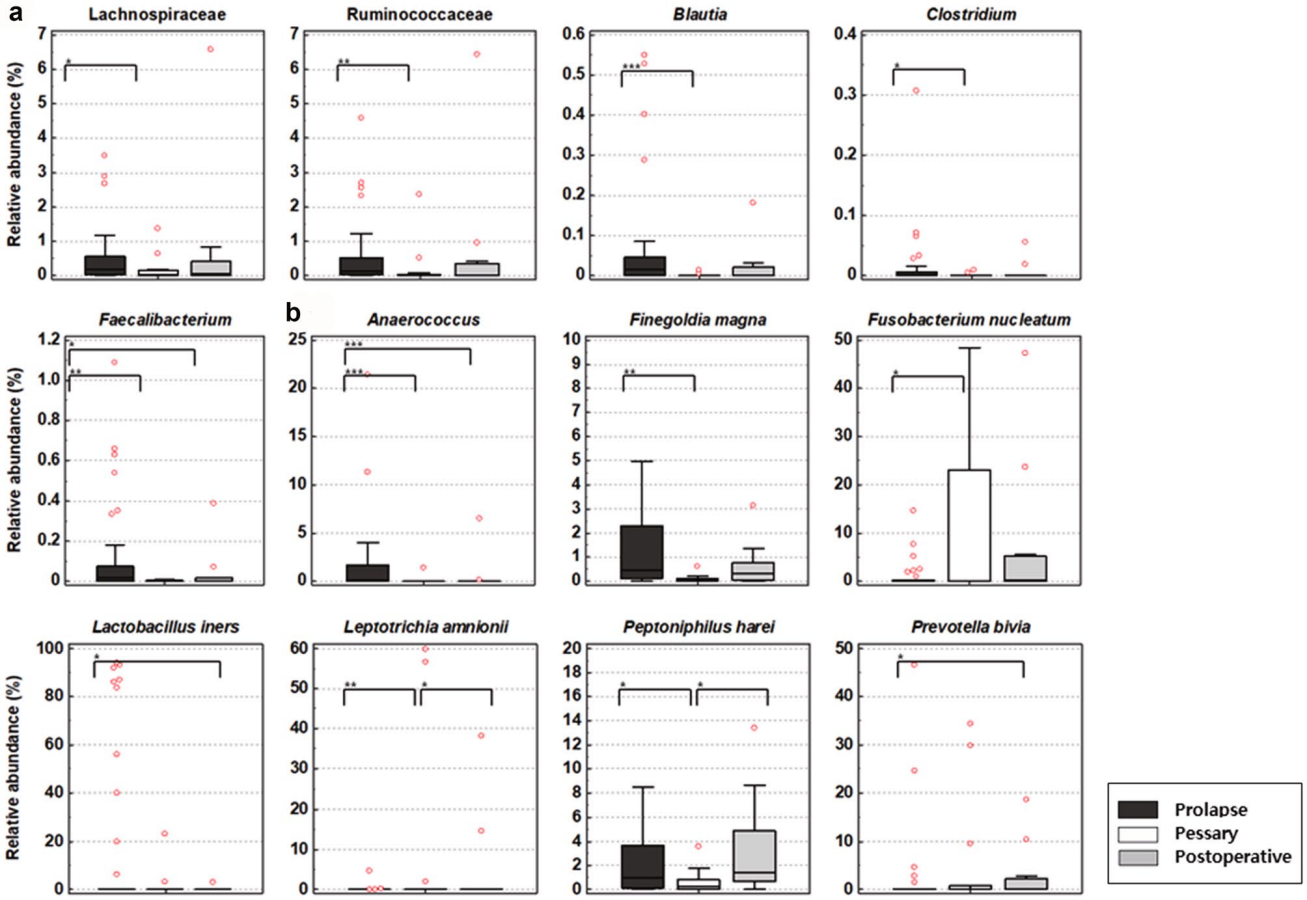
Relative abundance (%, y-axis) of the statistically significant taxa in the vaginal microbiota among three groups (x-axis): Prolapse (n = 42) vs. Pessary (n = 17) vs. Postoperative group (n = 11). (**a**) Relative abundance at the family or genus level of known important human gut taxa and (**b**) Relative abundance at the species level of 22 representative microbial taxa associated with vaginal health. Horizontal and box plots show the median and quartiles, respectively. Non-parametric test, ^*^p < 0.05, ^**^p < 0.01, and ^***^p < 0.001.

To estimate the contamination of human gut taxa in vaginal MTP in each group, we compared the relative abundance of 11 human gut taxa which are generally known to be important in human gut through many previous studies^10,11^ (family Christensenellaceae, Enterobacteriaceae, Lachnospiraceae, and Ruminococcadeae; genera *Akkermansia*, *Bacteroides*, *Blautia*, *Campylobacter*, *Clostridium*, *Escherichia*, and *Faecalibacterium*). Among them, the proportions of Lachnospiraceae, Ruminococcaceae, *Blautia*, *Clostridium* and *Faecalibacterium* were significantly different (*p* = 0.0187, 0.0053, 0.0006, 0.0466, and 0.0014, respectively), and all were higher in the prolapse group (Fig. 4a). The relative abundance of the 22 representative microbial taxa, associated with vaginal health revealed that *Anaerococcus*, *Finegoldia magna*, *Fusobacterium nucleatum*, *Lactobacillus iners*, *Leptotrichia amnionii*, *Peptoniphilus harei*, and *Prevotella bivia* were significantly different among the three groups (*p* < 0.0001, 0.0019, 0.0251, 0.0399, 0.0039, 0.0355, and 0.0480, respectively) (Fig. 4b). Interestingly, there were differences in the dominant bacterial vaginitis species *Anaerococcus*, *F. magna* and *L. iners* were high in the prolapse group; *F. nucleatum* and *L. amnionii* were high in the pessary group; *P. harei* and *P. bivia* were high in the postoperative group. However, the abundance of *Atopobium vaginae*, *Gardnerella vaginalis* and *Prevotella timonensis* were not statistically different among the three groups (*p* = 0.9039, 0.3426, and 0.0907, respectively, by Kruskal–Wallis test). These results indicate that contamination by gut microbiota in POP patients was reduced after pessary or surgery, whereas the dominant vaginitis-related bacteria changed after treatment rather than definitely reduced.

## Discussion

In the present study, we investigated and compare the vaginal microbiome of POP patients, pessary treated patients, and patients treated with reconstructive operation. Yoshimura et al.^12^ study showed that patients with pessary therapy with *Lactobacillus* did not always disturb intravaginal microbial flora. However, patients with erosions with pessary displayed significant differences in their vaginal microbiota, which contained a much greater bacterial diversity with an increase in gram-negative bacteria and a decrease in lactobacilli^13^. Alnaif and Drutz^14^ reported an increased risk of bacterial vaginosis in pessary users and suggested that shifts in vaginal flora may be associated with the development of vaginal erosion, which is consistent with our findings. There is no comparison study of the vaginal microbiome in the healthy group, the POP group and pessary group yet.

The vaginal microbial diversity differed among the three groups. The POP group showed the highest species abundance, and the taxonomic composition distribution was different from the pessary and postoperative groups. In particular, the distribution of CST IV was higher than other CST in the prolapse group, and there was no statistical difference in microbiome diversity between different stages of POP (stage I, II, III, IV).

Interestingly, CST IV was only observed in pessary and postoperative groups indicating that this result suggests that both treatment did not have any effect on the vaginal microbiome. The sample sizes of the three groups are significantly different, with CST IV being the primary concentration. Perhaps the occurrence of the lactobacilli-dominant CST is a random event, or maybe the recovery of lactobacilli takes time. Therefore, long-term follow-up studies are needed to track changes over an extended period. Short-term assessments might not find the complete picture of how the microbiome responds and evolves post-treatment.

The study found that after the anatomy was restored through either pessary or operation treatment, there was a reduction in contamination by gut microbiota in the vagina. Although the treatment may have been successful in restoring the anatomy and reducing gut microbiota contamination, it may not have been effective in eliminating vaginitis-related bacteria, which can cause vaginal infections and other vaginal health issues.

According to Amabebe and Anuroba^15^, lactobacilli-dominant microbiota confer an anti-inflammatory environment, resulting from the production of lactic acid, which stimulates the production of large amounts of anti-inflammatory cytokines which in turn inhibits pathogen attachment to the vaginal epithelium and decreases the risk of other infections. *Lactobacillus crispatus*-dominated vaginal communities were associated with lower levels of inflammation-related cytokines (e.g., IL-1α, IL-1β, and IL-8) compared to communities dominated by BV-associated bacteria or *Lactobacillus iners*^16,17^. There were no cytokines that were significantly different between CST groups in this study, which should be taken into account that the number of CST group IV is high and the number of other groups is very small, which may make statistical comparisons imprecise. Also, species-level annotation based on 16 s rRNA sequencing may not achieve the accuracy required for CST grouping. Interestingly, the use of pessary led to an increase in cytokines such as IFN-γ, IL-1β, IL-4, IL-10, IL-12p70, and TNF-α, which suggests that pessary use may lead to increased inflammation. Additionally, even with anatomical restoration, the risk of inflammation (vaginal discharge, bleeding, and erosion)^7^ still appeared to increase due to the foreign body effect.

Probiotic therapy has been reported to be useful in maintaining normal intravaginal microbial flora. Studies have shown that certain strains of probiotics, such as *Lactobacillus* species and its multi-microbial interaction can help restore and maintain a healthy balance of bacteria in the vagina^18,19^. The probiotic activity and protective effects attributed to lactobacilli in the vaginal microbiota are influenced by several factors, including the composition and diversity of the microbial consortium present^20^. The effectiveness of lactobacilli in promoting vaginal health is not solely determined by their total number but also by the specific species and strains of lactobacilli present^19,20^. Different *Lactobacillus* species can have varying effects on vaginal health, and certain strains might be more effective at promoting a balanced microbiota and preventing infections^21,22^.

Estrogen therapy is known to help improve the vaginal microbiome in postmenopausal women^21^, there has been no randomized controlled trial analyzing the benefit of intravaginal estrogen use in women with pessaries^23^. It has been reported that the use WO 3191 pessaries (contained the amphoteric tenside cocoamphopropionate) and, like lactic acid pessaries (contained lactic acid and sodium lactate) prevent recurrence of bacterial vaginosis ^24^. These pessary were not able to prevent recurrent bacterial vaginosis, and the health-associated *Lactobacillus* sp. such as *L, crispatus* is more important to maintain vaginal microbiome stabilization^24^. Vaginal microbiota dysbiosis, characterized by the loss of *Lactobacillus* dominance and increase of microbial diversity, was observed in the POP patients, and is closely related to gynecological diseases^24^. Therefore, improving the vaginal distribution of *Lactobacillus* species such as *L. crispatus*, and restoring the vaginal microbiome composition in POP patients is important in preventing further POP-induced gynecological diseases.

Our study is significant in that it is the first to compare the vaginal microbiome among POP and anatomical restorative intervention patients, and we expect that our findings contribute to the understanding of microbiome environment in POP and anatomical restorative intervention patients.

There are a few limitations in this study: firstly, the sample size of pessary (n = 17) and postoperative groups (n = 11) were small compared to POP group (n = 42), and is designed as a cross-sectional study, and the samples are not from the same patient before and after treatment. Actually, the sample of our study was determined based on the availability of eligible participants during the study period. Secondly, defining stage of prolapse can be subjective, and there were many cases without a diagnosis even in the presence of symptoms. Therefore, further prospective research is needed to determine the impact and change of POP on the vaginal microbiome.

Treatment (pessary insertion or reconstructive operation) for POP can lead to resolution of structural discomfort. However, there is also evidence to suggest that these treatments may not fully restore the balance of the vaginal microbiome. Therefore, it is important to address any imbalances in the vaginal microbiome as part of the overall treatment plan to prevent additional gynecological diseases.

## Materials and methods

### Study design and participants

The study was approved by the Institutional Scientific and Ethics Committee (protocol number: HC20TISIO100). All patients with POP who visited the Department of Obstetrics and Gynecology in university hospital, between October 2020 and February 2022, were recruited for the study. Informed consent was obtained and participants were screened to confirm their eligibility before enrollment and all methods were carried out in accordance with relevant guidelines and regulations.

**Figure 5:**
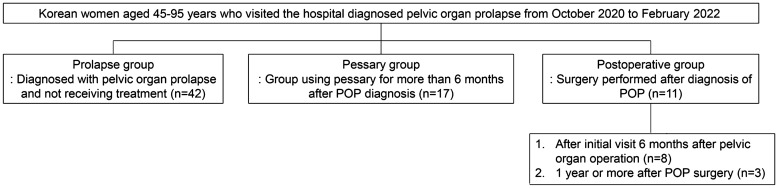
Diagram showing the number of patients within each study group (prolapse, pessary, and postoperative).

Women using any antibiotics, vaginal moisturizers, or vaginal tablets were excluded, since these products affect the vaginal microbiota. Seventy participants, between 45 and 95 years of age, were enrolled. Participants were categorized into three groups for the cross-sectional study, (a) Prolapse group (n = 42): patients diagnosed with POP, using the International Continence Society approved Pelvic Organ Prolapse Quantification (POP-Q) system^25^, and monitored without treatment; (b) Pessary group (n = 17): POP patients treated with pessary insertion and monitored for more than 6 months; (c) Postoperative group (n = 11): POP patients treated with pelvic reconstructive operation (laparoscopic-assisted vaginal total hysterectomy with anteroposterior colpoperineorrhaphy and uterosacral ligament suspension) and monitored for more than six months (Fig. 5).

### Participant data and vaginal sampling

Patient characteristics, medical and/or surgical histories were recorded. Standardized pelvic examination was performed using a sterile speculum in the lithotomy position. A physician collected participant samples from the posterior fornix of the vaginal wall, using a flocked swab (NFS-2 swab; Noble Biosciences, Inc., Whaseong-si, Korea) which was placed in 1 mL of phosphate-buffered saline and stored at − 80 °C. In case pelvic organ bulging, reduction was performed intravaginally, and for the pessary group, examination was carried out in the presence of the pessary.

### Multiplex immunoassay

For cytokine analysis, vaginal swab samples were thawed on ice, vortexed for approximately 10 s and centrifuged at 2500 rpm for 10 min. Sample supernatant was transferred into a new microcentrifuge tube and sample cytokines (n = 10: IFN-γ, IL-10, IL-12p70, IL-1β, IL-2, IL-4, IL-6, IL-8, MIP-1b, and TNFα) were quantified using a MILLIPLEX MAP Human Cytokine/Chemokine Magnetic Bead-based multiplex immunoassay (Millipore, Billerica, MA) for. Cytokine/chemokine levels were measured with the FLEXMAP 3D instrument and analyzed using xPONENT Software V4.2 for Lumniex 200 system (Luminex Corporation, Austin, USA).

### DNA extraction and 16S rRNA gene sequencing

For DNA extraction, vaginal swab samples in 1 mL nucleic acid preservation media were thawed on ice. Subsequently, vortexed for approximately 10 s and centrifuged at 2500 rpm for 10 min and supernatants were transferred to new tubes. The 450uL supernatant was added to 300 µL isopropanol and vortexed for 30 s. The mixture was placed in a DNeasy Mini spin column from the Qiagen DNeasy Blood and Tissue kit (QIAGEN, Venlo, Netherlands) and DNA extraction was performed according to the manufacturer's protocol. DNA concentration and purity were measured using a spectrophotometer (Eppendorf D30 (Eppendorf, Hamburg, Germany)). DNA concentration of more than or equal to 15 ng/μl with A260/A280 ratio of 1.8–2.0 was used for 16S rRNA sequencing. The genomic DNA was amplified by PCR, using fusion primers 341F (5′-AATGATACGGCGACCACCGAGATCTACAC-XXXXXXXX-TCGTCGGCAGCGTC-AGATGTGTATAAGAGACAG-CCTACGGGNGGCWGCAG-3′) and 805R (5′-CAAGCAGAAGACGGCATACGAGAT-XXXXXXXX-GTCTCGTGGGCTCGG-AGATGTGTATAAGAGACAG-GACTACHVGGGTATCTAATCC-3′; underlining sequence indicates the complementary region of the primer) binding to the V3–V4 regions of the 16S rRNA gene, as previously reported^26^.

### Bioinformatics analyses

The raw sequence reads were pre-processed for quality checks, including trimming of low-quality amplicons, diversity calculation, comparative microbiome taxonomic profile (MTP) analysis, and taxonomic biomarker discovery analysis, using an EzBioCloud App (CJ Bioscience, Inc., Seoul, Korea), as previously reported^26^. Alpha diversity indices were calculated using ACE and Chao1 for species richness, and Shannon and NPShannon to measure species diversity and evenness, respectively. To visualize sample differences, beta diversity distances were calculated using principal coordinate analysis (PCoA) based on the Jensen-Shannon method. Beta set-significance analysis between two MTP sets was performed using the permutational multivariate analysis of variance (PERMANOVA) test. Taxonomic biomarkers were displayed using the Linear Discriminant Analysis (LDA) Effect Size (LEfSe)-based cladogram^23^.

### Statistical analyses

Non-parametric comparison tests (Kruskal–Wallis test for more than two groups and Mann–Whitney U-test for two groups) were used to compare MTP results using MedCalc version 20.114 (MedCalc Software, https://www.medcalc.org/, Ostend, Belgium). A *p-*value less than 0.05 was considered statistically significant.

### Supplementary Information

Supplementary Information.

## Data Availability

The 16S rRNA gene sequences have been submitted to the NCBI Sequence Read Archive SRA and are available under the BioProject ID PRJNA965912.
