# Supplementary material for: Microbiome alterations in women with pelvic organ prolapse and after anatomical restorative interventions
Source: Sci Rep. 2023 Oct 16;13:17547. doi: 10.1038/s41598-023-44988-6 (PMC10579314; doi:10.1038/s41598-023-44988-6)
Supplement: Supplementary file 1 — Supplementary Information. [file 41598_2023_44988_MOESM1_ESM.pdf]

Microbiome alterations in women with pelvic organ prolapse and after anatomical restorative interventions.

Myungshin Kim1†, Seungok Lee2†, Hoon Seok Kim1, Mi Yeon Kwon3, Jaeeun Yoo2, & Min Jeong Kim4*

[1]Departments of Laboratory Medicine, Seoul St. Mary’s Hospital, College of Medicine, The Catholic University of Korea, Seoul, Republic of Korea.
[2]Departments of Laboratory Medicine, Incheon St. Mary’s Hospital, College of Medicine, The Catholic University of Korea, Seoul, Republic of Korea.
[3]Departments of Clinical Medicine Research, Bucheon St. Mary's Hospital, The Catholic University of Korea, Seoul, Republic of Korea. [4]Departments of Obstetrics and Gynecology, Bucheon St. Mary’s Hospital, College of Medicine, The Catholic University of Korea, Seoul, Republic of Korea

Supplementary Table 1. Linear Discriminant Analysis Effect Size (LEfSe)-based cladogram showing the differential phylogenic distribution of the bacterial taxa related to the prolapse (red), pessary (blue), and post-operative (green) groups. The taxonomic names of the phyla are indicated; and class, order, family, and genus are abbreviated.

Abbreviation	Taxon name	Taxon rank	Taxonomy
	Bacteroidetes	Phylum	Bacteria
	Firmicutes	Phylum	Bacteria
	Fusobacteria	Phylum	Bacteria
a	Bifidobacteriaceae	Family	Bacteria: Actinobacteria: Actinobacteria_c: Bifidobacteriales
c	Corynebacterium	Genus	Bacteria: Actinobacteria: Actinobacteria_c: Corynebacteriales: Corynebacteriaceae
d	Corynebacteriaceae	Family	Bacteria: Actinobacteria: Actinobacteria_c: Corynebacteriales
e	Lawsonella	Genus	Bacteria: Actinobacteria: Actinobacteria_c: Corynebacteriales: Lawsonella_f
f	Lawsonella_f	Family	Bacteria: Actinobacteria: Actinobacteria_c: Corynebacteriales
g	Corynebacteriales	Order	Bacteria: Actinobacteria: Actinobacteria_c
h	Brevibacterium	Genus	Bacteria: Actinobacteria: Actinobacteria_c: Micrococcales: Brevibacteriaceae
i	Brevibacteriaceae	Family	Bacteria: Actinobacteria: Actinobacteria_c: Micrococcales
j	Propionibacteriaceae	Family	Bacteria: Actinobacteria: Actinobacteria_c: Propionibacteriales
k	Propionibacteriales	Order	Bacteria: Actinobacteria: Actinobacteria_c
l	Coriobacteriaceae	Family	Bacteria: Actinobacteria: Coriobacteriia: Coriobacteriales
m	Coriobacteriales	Order	Bacteria: Actinobacteria: Coriobacteriia
n	Coriobacteriia	Class	Bacteria: Actinobacteria
o	Prevotella	Genus	Bacteria: Bacteroidetes: Bacteroidia: Bacteroidales: Prevotellaceae
p	Prevotellaceae	Family	Bacteria: Bacteroidetes: Bacteroidia: Bacteroidales
q	Bacteroidales	Order	Bacteria: Bacteroidetes: Bacteroidia
r	Bacteroidia	Class	Bacteria: Bacteroidetes
s	Staphylococcus	Genus	Bacteria: Firmicutes: Bacilli: Bacillales: Staphylococcaceae
t	Staphylococcaceae	Family	Bacteria: Firmicutes: Bacilli: Bacillales
u	Bacillales	Order	Bacteria: Firmicutes: Bacilli
v	Facklamia	Genus	Bacteria: Firmicutes: Bacilli: Lactobacillales: Aerococcaceae
w	Aerococcaceae	Family	Bacteria: Firmicutes: Bacilli: Lactobacillales
x	Lactobacillales	Order	Bacteria: Firmicutes: Bacilli
y	Bacilli	Class	Bacteria: Firmicutes
z	Faecalibacterium	Genus	Bacteria: Firmicutes: Clostridia: Clostridiales: Ruminococcaceae
a0	Fastidiosipila	Genus	Bacteria: Firmicutes: Clostridia: Clostridiales: Ruminococcaceae
a1	Bulleidia	Genus	Bacteria: Firmicutes: Erysipelotrichi: Erysipelotrichales: Erysipelotrichaceae
a2	Erysipelotrichaceae	Family	Bacteria: Firmicutes: Erysipelotrichi: Erysipelotrichales
a3	Erysipelotrichales	Order	Bacteria: Firmicutes: Erysipelotrichi
a4	Erysipelotrichi	Class	Bacteria: Firmicutes
a5	Veillonella	Genus	Bacteria: Firmicutes: Negativicutes: Veillonellales: Veillonellaceae
a6	Veillonellaceae	Family	Bacteria: Firmicutes: Negativicutes: Veillonellales
a7	Veillonellales	Order	Bacteria: Firmicutes: Negativicutes
a8	Negativicutes	Class	Bacteria: Firmicutes
a9	Parvimonas	Genus	Bacteria: Firmicutes: Tissierellia: Tissierellales: Peptoniphilaceae
b	Bifidobacteriales	Order	Bacteria: Actinobacteria: Actinobacteria_c
b0	Fusobacterium	Genus	Bacteria: Fusobacteria: Fusobacteria_c: Fusobacteriales: Fusobacteriaceae
b1	Fusobacteriaceae	Family	Bacteria: Fusobacteria: Fusobacteria_c: Fusobacteriales
b2	Sneathia	Genus	Bacteria: Fusobacteria: Fusobacteria_c: Fusobacteriales: Leptotrichiaceae
b3	Fusobacteriales	Order	Bacteria: Fusobacteria: Fusobacteria_c
b4	Fusobacteria_c	Class	Bacteria: Fusobacteria
b5	Alphaproteobacteria	Class	Bacteria: Proteobacteria
b6	Citrobacter	Genus	Bacteria: Proteobacteria: Gammaproteobacteria: Enterobacterales: Enterobacteriaceae
